# Densities of Eggs and Nymphs and Percent Parasitism of *Bemisia tabaci* (Hemiptera: Aleyrodidae) on Common Weeds in West Central Florida

**DOI:** 10.3390/insects5040860

**Published:** 2014-11-10

**Authors:** Hugh A. Smith, Curtis A. Nagle, Gregory A. Evans

**Affiliations:** 1 University of Florida, IFAS, Gulf Coast Research and Education Center, 14625 C.R. 672, Wimauma, FL 33598, USA; E-Mail: cnagle@ufl.edu; 2 United States Department of Agriculture, 10300 Baltimore Avenue, BARC-West, Bldg 005, Rm. 09A, Beltsville, MD 20705, USA; E-Mail: gregory.a.evans@aphis.usda.gov

**Keywords:** *Encarsia*, *Eretmocerus*, sweetpotato whitefly, weed hosts, whitefly parasitism

## Abstract

The density of eggs and nymphs of *Bemisia tabaci* (Gennadius) biotype B and the percent parasitism of the nymphs were measured from specimens collected on nine species of weeds, commonly found in west central Florida during the spring and summer of 2012 and 2013. The weeds were direct seeded in 2012 and grown as transplants in 2013 for Randomized Complete Block design experiments. The leaf area of each whole-plant sample was measured and the *B. tabaci* density parameters were converted to numbers per 100 cm^2^. In June and July, 2013, whole-plant samples became too large to examine entirely, thus a representative portion of a plant totaling about 1000 cm^2^ was sampled. Egg and nymph densities and percent parasitism varied greatly among weed species, and were higher overall in 2012 than in 2013. The highest densities of eggs and nymphs were measured on *Abutilon theophrasti*, *Cassia obtusifolia* and *Emilia fosbergii* each year. Lower densities of immature *B. tabaci* were measured on most dates for *Amaranthus retroflexus*, *Bidens alba*, *Ipomoea lacunosa*, *Sesbania exaltata* and *Sida acuta*. Nymph to egg ratios of 1:4 were observed on *A. theophrasti* and *S. exaltata* in 2012, while less than one nymph per ten eggs was observed overall on *A. retroflexus*, *E. fosbergii* and *I. lacunosa*. In 2012, parasitism rates of 32.3% were measured for *B. alba*, 23.4% for *C. obtusifolia* and 17.5% for *S. acuta*. Of the 206 parasitoids reared out over two seasons, 96.6% were *Encarsia* spp. and the remainder *Eretmocerus* spp. The role of weeds in managing *B. tabaci* is discussed.

## 1. Introduction

*Bemisia tabaci* (Gennadius) biotype B (Hemiptera: Aleyrodidae), formerly known as *Bemisia argentifolii* (Bellows and Perring) or as the Middle East-Asia Minor 1 (MEAM1) genetic group of *Bemisia tabaci*, attacks a broad range of horticultural, ornamental and row crops [1]. The species causes damage through removal of sap, by producing honeydew, which serves as a substrate for sooty molds that reduce quality [2], and by causing crop disorders, such as irregular ripening of tomato [3]. It is known to transmit over 100 plant viruses in the families *Geminiviridae*, *Closteroviridae* and *Potyviridae* [4] including *Tomato yellow leaf curl virus* (TYLCV), which is the most important pest problem afflicting tomato production in Florida and many other tomato producing regions [5]. Florida is one of the foremost producers of fresh tomato in the United States [6]. *Bemisia tabaci* is the only whitefly pest of significance attacking tomato in Florida [7].

In addition to developing on many horticultural crops, *B. tabaci* can develop on many species of weeds [8,9]. Weeds may enhance *B. tabaci* problems by serving as hosts for *B. tabaci* near crops and between cropping seasons [10]. In addition, weeds may serve as alternate hosts for whitefly-transmitted viruses [11,12,13,14,15]. Surveys of weeds in Florida have not detected TYLCV in common weed hosts of *B. tabaci* [16]. However research in Latin America and the Mediterranean indicates that weeds species (including species present in Florida) are hosts of TYLCV [13,14,15]. Weeds have been identified as hosts for *Bemisia tabaci*-transmitted viruses affecting other horticultural crops in Florida, including bean golden mosaic, cucurbit leaf crumple and squash vein yellowing viruses [17].

Weeds may contribute to the regulation of populations of *B. tabaci* by providing a habitat for its predators and parasitoids [18,19,20,21]. Stansly *et al.* [20] quantified the densities of *B. tabaci* and its parasitoids on weeds in southwest Florida and found relatively high percentages of parasitism on *Bidens* sp. and *Lantana* sp. At least thirteen species of parasitoid have been reared from *B. tabaci* in Florida [22]. *Encarsia pergandiella* Howard (Hymenoptera: Aphelinidae), *Encarsia nigricephala* Dozier (Hymenoptera: Aphelinidae) and *Eretmocerus* spp. (Hymenoptera: Aphelinidae) predominated, comprising 62%, 17% and 12% respectively of species collected [23]. In west central Florida, tomatoes are transplanted in late winter/early spring (January–March) and late summer/early fall (August–October), with harvest carried out 90–120 days after transplanting, depending on weather and market conditions. Populations of *B. tabaci* in Florida typically decrease markedly during the cooler winter months, but can be sustained at high levels on alternate hosts and abandoned or improperly destroyed tomato fields during the climatically favorable months of summer. For this reason, it is important to characterize the importance of summer weeds as hosts of *B. tabaci*, and reservoirs of TYLCV and whitefly parasitoids.

Currently, research is being conducted at the University of Florida’s Gulf Coast Research and Education Center (GCREC) to determine the role of summer weeds in regards to the management of *B. tabaci* and TYLCV in the west central Florida tomato growing region. The weeds being evaluated include: *Abutilon theophrasti* Medik (Malik) (Malvaceae), *Amaranthus retroflexus* L. (Amaranthaceae), *Bidens alba* L. (Asteraceae), *Cassia* (*Senna*) *obtusifolia* L. (Irwin & Barneby) (Fabaceae), *Emilia fosbergii* Nicolson (Asteraceae), *Ipomoea lacunosa* L. (Convolvulaceae), *Sesbania exaltata* (Raf.) Cory (Fabaceae), *Sida acuta* Burm. F. (Malvaceae), and *Solanum americanum* Mill. (Solanaceae). These weeds, and in some cases other species in the same genera, are common on the edges and in irrigation ditches of agricultural fields in west central Florida as well as other parts of the state [24]. With the exception of *B. alba*, which is present year round, these weed species are most abundant during the spring and through the summer. In addition to being common in the field, *B. alba*, *E. fosbergii* and *S. acuta* are also common in and around the nurseries and screen houses where seedlings of tomato and other horticultural crops are produced.

The objective of the study was to improve understanding of the relative importance of these weeds as summer hosts for *B. tabaci* and its parasitoids. Large scale commercial production of tomato in Florida largely ceases during the hottest summer months, July and August. Therefore information on the relative importance of these weeds as alternate hosts of *B. tabaci* when crops are not present is of value. Here we report comparative information on the densities of *B. tabaci* eggs, nymphs and percent parasitized nymphs on key weeds. Weeds that are determined in the future to be non-hosts of TYLCV would essentially be “dead ends” for the virus. *Bemisia tabaci* developing on non-virus hosts will be virus-free. Therefore weeds which demonstrate a high percentage of parasitism and do not serve as reservoirs for TYLCV may play a positive role in the suppression of viruliferous *B. tabaci* populations.

## 2. Experimental Section

### 2.1. Field Study Establishment

*Abutilon theophrasti*, *Amaranthus retroflexus*, *Bidens alba*, *Cassia obtusifolia*, *Emilia fosbergii*, *Ipomoea lacunosa*, *Sesbania exaltata*, *Sida acuta*, and *Solanum americanum* were studied at the University of Florida, GCREC, Wimauma FL (N27°45.599', W82°13.446') during the spring and summer of 2012 and 2013. Seed of *A. theophrasti*, *C. obtusifolia* and *Sesbania exaltata* was purchased from Azlin Weed Seed Service (Leland, MS, USA). Seed of *A. retroflexus*, and *Ipomoea lacunosa* was purchased from V and J Seed Farms, Inc. (Woodstock, IL, USA). Seed of the remaining weed species in the study was collected from plants growing at GCREC. Weeds were direct seeded into the experimental plots on 27 April 2012 using 2–3 seeds per plant hole, and thinned to one plant per hole after germination. Because of differences in germination and growth rate of the direct seeded plants, comparisons between some host plants were difficult. To avoid this in 2013, each weed species was grown from seed in a growth room with 12:12 (L:D) h at 26–30 °C and then transplanted 10 April, three to four weeks post germination. By transplanting all weeds at the same phenological stage, side by side comparison was facilitated.

The weeds were maintained in 20 cm high and 81 cm wide beds of Myakka fine sand, spaced on 1.5 m centers, covered with white, virtually impermeable plastic mulch and irrigated with drip irrigation without the injection of liquid fertilizer. Each year the experiment was arranged in a Randomized Complete Block design. Each treatment consisted of different weed species replicated four times and planted in a single row of ten plants with 0.3 m between the plants. Plots were spaced 3 m apart, with an unplanted bed between the plots. The plants were infested naturally with *B. tabaci* from populations that occurred around the study site.

### 2.2. Sampling

In 2012, sampling began on 8 May and was carried out nearly every week through 17 July. Samples consisted of one whole plant per plot from each replication. Weed species emerged and grew at different rates, with the result that not all species were sampled on each sampling date. *A. theophrasti*, *C. obtusifolia*, *I. lacunosa*, and *S. exaltata* were sampled during the entire period. Sampling for *B. alba* was initiated 16 May; sampling for *A. retroflexus* and *E. fosbergii* began 25 May. *S. acuta* and *S. americanum* emerged weeks after other species and initially grew very slowly. These species were sampled on 11 and 17 July only. Plant samples were brought to the laboratory at GCREC and the underside of all leaves was examined using a dissecting microscope. From the 8th of May to the 7th of June, the number of eggs and nymphs of *B. tabaci* and the total leaf area per plant were recorded. Leaf area was measured with a LI-COR Portable Area Leaf Meter LI-3000 (LI-COR, Lincoln, NE, USA). By mid-season, most plants had become very large; therefore after the 7th of June, leaf area was not measured and only the number of non-parasitized and parasitized nymphs per plant was recorded. In 2013, sampling occurred from 17 April through 1 July. Numbers of eggs, non-parasitized and parasitized nymphs and leaf area per sample were recorded. When plants became too large for entire whole plant samples to be examined (3 June–1 July), a portion of the plant consisting of one third lower, one third mid and one third upper stratum foliage was selected. These later samples consisted of a total of approximately 1000 cm^2^ per plant.

Parasitized nymphs were observed on three sample dates in 2012 (2–17 July) and on six sample dates in 2013 (30 April–1 July). The focus of the late-season samples in both years was to determine the suitability of weeds as hosts for parasitoids by comparing the proportion of parasitized nymphs to non-parasitized nymphs on each species.

### 2.3. Parasitoid Collection

Leaves from a plant sample possessing parasitized nymphs were maintained on moistened filter paper in 60 × 15 mm petri dishes (Fisher Scientific, Waltham, MA, USA; cat. no. 08-757-13A) inside 1.4 liter food service containers (Sterilite Corporation, Ennis, TX, USA) in a growth room with 14:10 (L:D) h at 26–30 °C. Foliage with parasitized nymphs was placed in the food service container with moistened paper to maintain humidity and a yellow sticky card (Olson Products, Medina OH) on the inner cover to attract emerged parasitoids. Emerged parasitoids were removed from the sticky card using Histo-Clear (National Diagnostics, Atlanta, GA, USA) and were sent to the USDA lab in Beltsville MD for identification by Gregory A. Evans.

### 2.4. Statistical Analysis

Egg and nymph data were converted to number per 100 cm^2^ when a leaf area measurement accompanied the data and transformed using log_10_(x + 1) to meet assumptions of normality before analysis using PROC ANOVA with SAS 9.2 software [25]. Percent parasitized nymphs were transformed using arcsine [√(%x/100)] and analyzed similarly to the egg and nymph data.. All means were separated by Fisher’s Protected LSD test (*p* ≤ 0.05). Means are reported in the original scale.

## 3. Results and Discussion

### 3.1. 2012

There were significant differences in the densities of *B. tabaci* eggs observed across weed species from 16 May–11 June (Table 1). No egg data were recorded after 11 June.

**Table 1:** Mean (±SE) *B. tabaci* egg densities on selected weeds in 2012. Means within a column followed by the same letter are not significantly different (*p* < 0.05) by Fisher’s Protected LSD. Data were transformed log_10_(x + 1) prior to ANOVA; non-transformed means are presented.

*B. tabaci* Eggs per 100 cm^2^ Leaf Area (Mean ± SE)					
Weed Species	8 May	16 May	25 May	4 June	11 June
*A. retroflexus*	-	-	42.8^de^ (14.0)	40.1^c^ (3.9)	85.8^cd^ (21.2)
*A. theophrasti*	159.8^a^ (64.9)	540.6^a^ (225.5)	296.1^c^ (53.0)	328.5^a^ (105.4)	448.0^b^ (218.4)
*B. alba*	-	6.2^c^ (3.0)	28.4^ef^ (9.8)	48.7^c^ (5.7)	73.0^d^ (15.5)
*C. obtusifolia*	230.3^a^ (125.6)	70.7^b^ (25.3)	661.6^b^ (104.6)	262.3^ab^ (111.1)	402.9^ab^ (105.1)
*E. fosbergii*	-	-	1914.8^a^ (62.2)	292.9^a^ (13.3)	730.4^a^ (117.5)
*I. lacunosa*	133.3^a^ (34.0)	32.5^b^ (14.2)	64.7^d^ (11.2)	162.7^b^ (54.0)	152.3^c^ (23.6)
*S. exaltata*	59.3^a^ (35.2)	21.5^bc^ (6.3)	13.6^f^ (4.1)	11.1^d^ (4.0)	3.7^e^ (1.5)
*F*-value	1.00 (*F*_3,9_)	10.33 (*F*_4,12_)	61.58 (*F*_6,18_)	23.11 (*F*_6,18_)	44.60 (*F*_6,18_)
*p*-value	0.4375	0.0007	<0.0001	<0.0001	<0.0001

Egg densities were highest on *E. fosbergii*, *C. obtusifolia* and *A. theophrasti*. Expressed per 100 cm^2^ foliage, the highest egg densities measured on each species were 1914.8 ± 62.2 on *E. fosbergii* (25 May), 661.6 ± 104.6 on *C. obtusifolia* (25 May) and 540.6 ± 225.5 on *A. theophrasti* (16 May). Egg densities peaked on *I. lacunosa* at 162.7 ± 54, on *A. retroflexus* at 85.8 ± 21.2, and on *B. alba* at 73.0 ± 117.5. Egg densities on young *S. exaltata* were 59.3 ± 35.2/100 cm^2^ (8 May), but declined over subsequent weeks and were in the lowest group statistically 25 May–11 June.

Nymph densities overall were much lower than egg densities (Table 2). Nymph densities on *E. fosbergii* were highest on 25 May (64.6 ± 2.9 per 100 cm^2^) which was not statistically different from densities on *A. theophrasti*. Nymph densities on *E. fosbergii* declined over subsequent weeks, while densities on *A. theophrasti* and *C. obtusifolia* increased, reaching 180.1 ± 42.3 per 100 cm^2^ on *A. theophrasti* and 171.4 ± 60.2 on *C. obtusifolia* (not statistically different from each other). Densities on *B. alba* and *I. lacunosa* remained below 21 nymphs per 100 cm foliage and did not separate statistically on any sample date. Nymph densities were lowest on *A. retroflexus* and *S. exaltata*, not surpassing 6.8 ± 1.7 per 100 cm^2^ on any sample date.

**Table 2:** Mean (±SE) *B. tabaci* nymph densities on whole plants of selected weeds in 2012. Means within a column followed by the same letter are not significantly different (*p* < 0.05) by Fisher’s Protected LSD. Data were transformed log_10_(x + 1) prior to ANOVA; non-transformed means are presented.

*B. tabaci* Nymphs per 100 cm^2^ Leaf Area (Mean ± SE)				
Weed Species	16 May	25 May	4 June	11 June
*A. retroflexus*	-	1.1^d^ (0.9)	4.3^d^ (3.2)	5.9^d^ (2.2)
*A. theophrasti*	33.2^a^ (27.3)	60.4^ab^ (23.2)	166.8^a^ (31.8)	180.1^a^ (42.3)
*B. alba*	0.4^a^ (0.4)	4.2^cd^ (2.5)	9.4^cd^ (3.9)	10.3^cd^ (4.9)
*C. obtusifolia*	17.9^a^ (5.5)	17.5^b^ (3.2)	53.7^b^ (11.0)	171.4^a^ (60.2)
*E. fosbergii*	-	64.6^a^ (2.9)	22.8^bc^ (6.9)	38.3^b^ (4.4)
*I. lacunosa*	7.3^a^ (2.5)	6.7^c^ (2.7)	18.0^c^ (8.5)	20.6^bc^ (9.0)
*S. exaltata*	5.2^a^ (1.7)	2.2^cd^ (1.2)	6.8^cd^ (1.7)	6.6^d^ (5.4)
*F*_6,18_	2.68 (*F*_4,12_)	14.64	14.33	22.36
*p*-value	0.0834	<0.0001	<0.0001	<0.0001

### 3.2. 2013

Egg and nymph densities were much lower overall in 2013 than 2012. Egg densities were very low in all weed species prior to April 30 (<7 eggs per 100 cm^2^). Egg densities on *A. theophrasti*, *E. fosbergii* and *S. acuta* peaked between 15 May and 3 June (Table 3). The greatest egg densities were observed on *A. theophrasti* on 28 May (147.0 ± 47.7 per 100 cm^2^); the highest egg densities observed on both *E. fosbergii* and *S. acuta* were ~53 per 100 cm^2^ foliage. As in 2012, egg densities were highest on most dates on *A. theophrasti* and comparable on some dates on *E. fosbergii*. Egg densities on *C. obtusifolia* were low relative to these two species in 2013. Egg densities on *C. obtusifolia* peaked on 17 May (17.0 ± 4.3 per 100 cm^2^). Egg densities on *S. americanum* increased till 10 June, peaking at 28.5 ± 9.7 per 100 cm^2^. Unlike egg densities on other species that increased to a point then declined, densities on *A. retroflexus*, *B. alba* and *I. lacunosa* remained constant and low throughout the trial, similar to what was observed in 2012. Egg densities averaged less than 11 per 100 cm^2^ on *I. lacunosa*, less than 9 per 100 cm^2^ on *A. retroflexus*, and less than 4 per 100 cm^2^ on *B. alba*. Eggs were rarely observed on *S. exaltata* in 2013.

There were no statistical differences in the density of nymphs among weed species prior to May 28 (Table 4). Nymph densities were highest on *A. theophrasti* and *E. fosbergii* during most of the sample period (28 May–1 July). Nymph densities on *S. americanum* were not statistically different from densities on *A. theophrasti* or *E. fosbergii* on a number of sample dates between 28 May and 1 July. Nymph densities peaked on *S. acuta* on May 28 (33.9 ± 3.2 per 100 cm^2^) when they were not statistically different from densities on *A. theophrasti*, *E. fosbergii* or *S. americanum*. Nymph densities on *C. obtusifolia* also peaked on 28 May, averaging 16.1 per 100 cm^2^. Nymph densities on *A. retroflexus*, *B. alba* and *I. lacunosa* remained low (<6 per 100 cm^2^) throughout the trial.

**Table 3:** Mean (±SE) *B. tabaci* egg densities on whole plants of selected weeds in 2013. Means within a column followed by the same letter are not significantly different (*p* < 0.05) by Fisher’s Protected LSD. Data were transformed log_10_(x + 1) prior to ANOVA; non-transformed means are presented.

Weed Species	*B. tabaci* Eggs per 100 cm^2^ Leaf Area (Mean ± SE)						
	30 April	15 May	28 May	3 June	10 June	17 June	1 July
*A. retroflexus*	1.8^b^ (0.8)	6.4^cd^ (2.8)	3.0^d^ (1.0)	2.0^c^ (0.8)	8.8^cd^ (3.3)	4.6^bc^ (3.0)	-
*A. theophrasti*	1.3^bc^ (1.0)	72.1^a^ (20.6)	147.0^a^ (47.7)	99.6^a^ (28.8)	140.9^a^ (20.2)	31.5^a^ (6.9)	5.6^ab^ (2.1)
*B. alba*	0.1^c^ (0.1)	1.4^d^ (0.2)	1.2^d^ (0.6)	2.0^c^ (0.6)	3.4^de^ (1.3)	0.4^c^ (0.2)	0.7^cd^ (0.5)
*C. obtusifolia*	1.4^bc^ (0.8)	17.0^b^ (4.3)	6.3^cd^ (2.8)	2.6^c^ (0.6)	1.4^e^ (0.6)	0.4^c^ (0.2)	0.0d (0.0)
*E. fosbergii*	1.6^b^ (0.3)	9.6^bc^ (5.7)	17.3^bc^ (4.3)	52.3^a^ (14.1)	44.8^b^ (11.2)	6.3^b^ (4.0)	14.1^a^ (6.9)
*I. lacunosa*	0.2^c^ (0.1)	10.2^bc^ (3.1)	6.5^cd^ (3.0)	6.1^c^ (4.2)	4.0^de^ (1.7)	0.4^c^ (0.2)	0.4^cd^ (0.3)
*S. acuta*	5.6^a^ (1.5)	52.9^a^ (14.9)	28.1^b^ (2.2)	17.4^b^ (2.5)	28.2^bc^ (16.9)	4.1^b^ (1.3)	1.8^cd^ (1.1)
*S. americanum*	0.6^bc^ (0.2)	8.8^bc^ (3.6)	7.3^cd^ (4.0)	19.6^b^ (7.9)	28.5^b^ (9.7)	7.2^b^ (3.3)	2.2^bc^ (0.8)
*F*_7,21_	5.24	10.23	9.76	17.13	21.26	9.19	8.19 (*F*_6,18_)
*p*-value	0.0014	<0.0001	<0.0001	<0.0001	<0.0001	<0.0001	0.0002

**Table 4:** Mean (±SE) *B. tabaci* nymph densities on whole plants of selected weeds in 2013. Means within a column followed by the same letter are not significantly different (*p* < 0.05) by Fisher’s Protected LSD. Data were transformed log_10_(x + 1) prior to ANOVA; non-transformed means are presented.

Weed Species	*B. tabaci* Nymphs per 100 cm^2^ Leaf Area (Mean ± SE)						
	30 April	15 May	28 May	3 June	10 June	17 June	1 July
*A. retroflexus*	0.1^a^ (0.1)	0.8^a^ (0.4)	4.9^c^ (1.3)	2.2^d^ (0.2)	3.6^c^ (1.1)	5.9^cd^ (0.7)	-
*A. theophrasti*	0.9^a^ (0.9)	12.7a (11.0)	50.2^ab^ (42.3)	47.4^a^ (7.1)	23.8^ab^ (10.2)	58.6^a^ (14.4)	12.5^a^ (0.8)
*B. alba*	0.1^a^ (0.0)	0.3^a^ (0.1)	4.4^c^ (0.5)	2.4^d^ (0.7)	2.5^c^ (0.4)	4.2^c–e^ (1.6)	2.9^bc^ (0.9)
*C. obtusifolia*	0.4^a^ (0.3)	2.1^a^ (0.8)	16.1^b^ (3.7)	7.6^c^ (2.6)	3.1^c^ (1.1)	3.1^de^ (0.5)	0.6^c^ (0.2)
*E. fosbergii*	0.3^a^ (0.1)	1.6^a^ (0.6)	49.5^a^ (7.8)	42.4^a^ (5.9)	41.4^a^ (6.8)	31.3^ab^ (9.5)	25.8^a^ (10.7)
*I. lacunosa*	0.1^a^ (0.1)	0.9^a^ (0.2)	4.5^c^ (2.2)	2.2^d^ (1.5)	2.4^c^ (1.0)	1.4^e^ (0.2)	0.5^c^ (0.4)
*S. acuta*	0.6^a^ (0.4)	5.3^a^ (1.5)	33.9^ab^ (3.2)	15.5^b^ (1.2)	15.5^b^ (6.7)	9.4^c^ (2.3)	2.2^c^ (0.3)
*S. americanum*	0.0^a^ (0.0)	0.7^a^ (0.1)	20.1^ab^ (6.6)	11.5^bc^ (1.3)	17.8^b^ (4.6)	34.1^b^ (14.1)	8.5^ab^ (3.2)
*F*_7,21_	0.64	2.05	6.38	27.82	11.54	18.77	9.34 (*F*_6,18_)
*p*-value	0.7199	0.0954	0.0004	<0.0001	<0.0001	<0.0001	<0.0001

**Table 5:** Mean (±SE) *B. tabaci* egg and nymph densities and nymph:egg ratio averaged over samples in 2012 and 2013. Means within a column followed by the same letter are not significantly different (*p* < 0.05) by Fisher’s Protected LSD. Data were transformed log_10_(x + 1) prior to ANOVA; non-transformed means are presented.

Weed Species	8 May–11 June, 2012			15 May–10 June, 2013		
	*B. tabaci* per 100 cm^2^ (Mean ± SE)		Nymphs per Egg (Mean ± SE)	*B. tabaci* per 100 cm^2^ (Mean ± SE)		Nymphs per Egg (Mean ± SE)
	Eggs	Nymphs		Eggs	Nymphs	
*A. retroflexus*	56.2^d^ (10.3)	3.8^d^ (1.1)	0.07^bc^ (0.02)	5.1^d^ (1.7)	2.9^d^ (0.4)	0.68^cd^ (0.15)
*A. theophrasti*	354.6^b^ (66.0)	88.1^a^ (16.6)	0.25^a^ (0.00)	114.9^a^ (20.2)	33.5^a^ (13.0)	0.29^e^ (0.09)
*B. alba*	39.1^d^ (7.2)	6.1^cd^ (1.7)	0.15^a–c^ (0.03)	2.0^e^ (0.5)	2.4^d^ (0.3)	1.34^a^ (0.24)
*C. obtusifolia*	325.6^b^ (54.5)	52.1^ab^ (12.9)	0.16^ab^ (0.03)	6.8^d^ (0.4)	7.2^c^ (1.2)	1.11^ab^ (0.26)
*E. fosbergii*	979.4^a^ (43.3)	41.9^b^ (2.7)	0.04^c^ (0.00)	31.0^b^ (3.4)	33.7^a^ (2.7)	1.11^ab^ (0.11)
*I. lacunosa*	109.1^c^ (5.3)	10.5^c^ (3.2)	0.09^bc^ (0.02)	6.7^d^ (1.3)	2.5^d^ (0.6)	0.37^de^ (0.04)
*S. acuta*	-	-	-	31.6^b^ (7.0)	17.5^b^ (2.1)	0.61^cd^ (0.09)
*S. americanum*	-	-	-	16.0^c^ (1.7)	12.5^b^ (1.8)	0.80^bc^ (0.11)
*S. exaltata*	21.8^e^ (7.6)	4.2^d^ (1.4)	0.25^a^ (0.11)	-	-	-
*F*_6,18_	73.46	35.53	4.31			
*F*_7,21_				63.07	41.50	9.16
*p*-value	<0.0001	<0.0001	0.0072	<0.0001	<0.0001	<0.0001

### 3.3. Nymph to Egg Ratios

There were significant differences among weed species in the ratio of nymphs to eggs each season (Table 5). In 2012, the highest nymph to egg ratio was observed in *A. theophrasti* and *S. exaltata* (1:4), followed by *B. alba* and *C. obtusifolia* (about 1: 6.5). Less than one nymph per ten eggs was observed in *A. retroflexus*, *E. fosbergii* and *I. lacunosa*. In 2013, overall numbers of eggs and nymphs were much lower across weed species compared to 2012. However the ratio of nymphs to eggs tended to be higher, indicating a higher proportion of surviving nymphs relative to the number of eggs. Densities of nymphs on *B. alba*, *C. obtusifolia*, and *E. fosbergii* were slightly higher than egg densities from May 15 to June 10. Female *B. tabaci* may have responded to senescing of the other host plants during this time period by ovipositing fewer eggs on these weeds, resulting in the highest nymph to egg ratio on these three species in 2013.

To determine if differences in nymph densities across weed species were related to differences in the number of nymphs successfully completing development, data were collected in 2013 on the number of *B. tabaci* nymphs in first, mid (2nd–3rd) and fourth instars. (The previous year we simply recorded nymph numbers without regard to instar.) Fourth instar nymphs were first observed on *B. alba* and *S. acuta* on 30 April, and on all other weeds except *A. retroflexus* and *A. theophrasti* by 15 May. Fourth instar nymphs were observed on *A. retroflexus* and *A. theophrasti* by 28 May. Exuviae were also observed on each weed species, but were in some cases too damaged to determine if they presented an adult *B. tabaci* exit hole, a parasitoid exit hole, or if they had been fed upon by a predator. For this reason the fourth instar was used as evidence that a certain percentage of nymphs were completing their life cycle on the host. Overall, the percentage of fourth instar nymphs was not statistically different among weed species when nymph counts from all sample dates are pooled (Table 6). This suggests that the proportion of nymphs completing development was similar for each weed species. The overall percentage of fourth instar nymphs was generally low for most species, ranging from around 4% for *A. theophrasti* and *S. americanum* to around 10 percent for *A. retroflexus*, *B. alba* and *C. obtusifolia.*

**Table 6:** Mean (±SE) *B. tabaci* non-parasitized nymphs, 4th instar nymphs and % 4th instar nymphs per sample in 20table13. Means within a column followed by the same letter are not significantly different (*p* *<* 0.05) by Fisher’s Protected LSD. Percent 4th instars were transformed arcsine [√(x/100)], other data were transformed log_10_(x + 1) prior to ANOVA; non-transformed means are presented.

Weed Species	Average of 24 Apr–1 July		
	*B. tabaci* per Sample (Mean ± SE)		% 4th Instars
	1st–4th Instars	4th Instars	(Mean ± SE)
*A. retroflexus*	11.6^d^ (3.6)	1.4^d^ (0.5)	10.35^a^ (4.31)
*A. theophrasti*	205.3^ab^ (37.5)	11.7^b^ (4.8)	3.85^a^ (1.36)
*B. alba*	24.7^c^ (4.9)	2.9^c^ (0.7)	10.31^a^ (1.26)
*C. obtusifolia*	144.2^b^ (71.2)	7.6^b^ (2.2)	10.28^a^ (1.80)
*E. fosbergii*	275.8^a^ (23.3)	26.6^a^ (4.7)	7.39^a^ (2.00)
*I. lacunosa*	18.8^cd^ (2.7)	1.9^cd^ (0.6)	8.60^a^ (3.29)
*S. acuta*	94.8^b^ (14.2)	7.8^b^ (2.6)	5.96^a^ (0.65)
*S. americanum*	140.5^ab^ (35.2)	7.4^b^ (1.6)	4.40^a^ (0.69)
*F*_6,18_	19.69	22.59	1.61
*p*-value	<0.0001	<0.0001	0.1863

### 3.4. Parasitism

Because of slow germination and growth, *S. acuta* and *S. americanum* were only sampled on 11 and 17 July 2012. By contrast, all *I. lacunosa* plants had senesced in 2012 before parasitism was observed. Higher levels of parasitism were observed for each species in 2012 than 2013 with the exception of *S. americanum*, which supported about 1% parasitism each year (Table 7). The highest percent of parasitism in 2012 was observed on *B. alba* (32.3%), followed by *C. obtusifolia* (23.4%) and *S. acuta* (17.5%) when sample dates in July were pooled. The highest percent parasitism observed for these species on a given week in 2012 was 58.6 (±15.9) % for *B. alba* (11 July), 36.8 (±17.6) for *C. obtusifolia* (2 July) and 28.8 (±24.0) % for *S. acuta* (17 July). In 2013, there were no statistical differences among weed species with regard to percent parasitism, which ranged from 0.4% in *A. theophrasti* to 2.8% in *C. obtusifolia*. The highest percent parasitism measured on a given week in 2013 was 14.6% on *C. obtusifolia* on 10 June, which was not statistically different from parasitism on *E. fosbergii* (7.8%) or *S. americanum* (7.2%) (*F*_7,21_ = 5.72, *p* = 0.0008). Percent parasitism was never greater than 10% on any given week for *I. lacunosa* in 2013. It was never greater than 7% on any week for *S. acuta* or 6% for *B. alba* that year. Percent parasitism was consistently less than 2% for *A. retroflexus* and *A. theophrasti* in 2013.

Of the 206 parasitoids reared out over the two seasons, 199 (96.6%) were *Encarsia* spp. and less than 4% were *Eretmocerus* spp (Table 8). Fourteen percent of *Encarsia* were identified as *E. sophia* and 12.5% were identified as *E. tabacivora.*

## 4. Conclusions

### 4.1. Colonization of Weed Hosts

Densities of *B. tabaci* were generally lower in 2013 than in 2012. A cause for the apparent differences between the two trials may have been weather conditions. The average temperature in May of 2013 was 23 °C, which as 2° C lower than that in May of 2012. [26]. Total rainfall for April and May were 3.4 cm and 4.7 cm respectively in 2012 compared to 10.6 cm and 9.0 cm for the same months in 2013.

Exposure to intense rain events can reduce whitefly populations [27] but non-severe rain events do not typically increase mortality of *B. tabaci* compared to *B. tabaci* protected conditions [28]. We do not expect that the increase in rain alone in 2013 reduced populations relative to 2012. Rather, plant establishment conditions in 2013 were generally cooler and wetter than in 2012, and we suspect that this slowed the build-up of *B. tabaci* populations during the second season. In 2013, all weed seedlings were transplanted into the field on the same day at a similar phenological stage to avoid the varied development times that occurred when weeds were grown from seed in the field in 2012. Most weeds were in the field for similar numbers of weeks each year, so we do not believe the overall difference in *B. tabaci* numbers in the two years was due to differences in exposure time to the pest.

**Table 7:** Mean (±SE) *B. tabaci* nymph densities and % parasitism on whole plants of selected weeds in 2012 and 2013. Means within a column followed by the same letter are not significantly different (*p* *<* 0.05) by Fisher’s Protected LSD. Nymph densities were transformed, log_10_(x + 1), and % parasitism transformed arcsine [√ (%x/100)] prior to ANOVA; non-transformed means are presented.

Weed Species	Mean *B. tabaci* Nymphal Densities Averaged over Samples & % Parasitism (Mean ± SE)					
	-------------------- 2012, July (per Plant) --------------------			------------- 2013, April-July (per Plant) -------------		
	Total	Parasitized	% Parasitism	Total	Parasitized	% Parasitism
*A. retroflexus*	3.7^d^ (1.6)	0.3^d^ (0.2)	7.9^cd^ (6.5)	13.5^d^ (4.2)	0.2^c^ (0.1)	1.1^ab^ (0.7)
*A. theophrasti*	16.9^cd^ (8.9)	0.4^cd^ (0.3)	6.2^cd^ (4.0)	235.4^ab^ (43.3)	1.2^bc^ (1.1)	0.4^b^ (0.4)
*B. alba*	37.6^bc^ (7.3)	11.3^a^ (1.8)	32.3^a^ (5.6)	28.6^c^ (5.8)	0.6^bc^ (0.2)	2.1^a^ (0.5)
*C. obtusifolia*	124.5^ab^ (51.9)	19.6^a^ (4.2)	23.4^ab^ (7.2)	166.9^b^ (81.7)	2.2^b^ (0.5)	2.8^a^ (1.7)
*E. fosbergii*	119.5^ab^ (71.9)	3.7^b^ (0.8)	6.8^b–d^ (2.5)	322.5^a^ (25.5)	7.6^a^ (1.2)	2.5^a^ (0.6)
*I. lacunosa^a^*	-	-	-	22.0^cd^ (3.3)	0.5^c^ (0.2)	2.2^a^ (0.6)
*S. acuta*	14.8^cd^ (4.9)	3.4^b–d^ (2.1)	17.5^a–c^ (7.3)	109.6^b^ (17.0)	1.5^bc^ (0.9)	1.1^ab^ (0.6)
*S. americanum*	243.0^a^ (43.3)	2.9^bc^ (1.6)	1.0^d^ (0.4)	161.9^ab^ (40.1)	1.4^bc^ (0.5)	1.1^ab^ (0.6)
*F*_6,18_	10.20	12.09	4.02	19.86 (*F*_7,21_)	6.95 (*F*_7,21_)	1.83 (*F*_7,21_)
*p*-value	<0.0001	<0.0001	0.0100	<0.0001	0.0002	0.1344

^a^ *I. lacunosa* had senesced before parasitism was observed.

**Table 8:** Parasitoids reared from *Bemisia tabaci* on weeds at GCREC, Balm, Florida in 2012 and 2013.

Weed Species	Parasitoid Taxa and No. of Specimens Identified					
	*Encarsia* spp.	*E.* *citrella*	*E.* *luteola*	*E. sophia*	*E. tabacivora*	*Eretmocerus* spp.
*A. retroflexus*	0	-	1	1	2	2
*A. theophrasti*	19	-	1	4	10	2
*B. alba*	36	1	-	6	-	-
*C. obtusifolia*	55	-	-	11	9	3
*E. fosbergii*	6	-	-	3	4	-
*I. lacunosa*	22	1	-	1	-	-
*S. acuta*	1	-	-	-	-	-
*S. americanum*	-	-	-	2	-	-
Total	142	2	2	28	25	7

In 2012, egg densities were highest on *A. theophrasti*, *C. obtusifolia* and *E. fosbergii* relative to other weed hosts. Egg densities tended to be highest on these three weeds in 2013 also, although not to the same degree as in the previous year. Choice studies have demonstrated that *Bemisia tabaci* will preferentially settle on and colonize some weed hosts in greater numbers than others [18,29,30]. Once the host plant has been accepted, oviposition by *Bemisia tabaci* is influenced by a number of host plant characteristics including type and density of trichomes, leaf waxiness, and secondary plant compounds, as well as the nutritional status and age of the plant [31]. Densities of several hundred eggs per 100 cm^2^ measured during some weeks on these hosts are comparable to densities measured on favored economic hosts such as cantaloupe [32].

Egg densities on *A. theophrasti*, *C. obtusifolia* and *E. fosbergii* were also high relative to nymph densities. Nymph to egg ratios may vary on different species because of a number of factors. Gachoka *et al.* [30] observed that percent egg hatch of *B. tabaci* varied significantly among different weed species, ranging from as low as 0% on *A. retroflexus* and *Malvastrum coromandelianum* L. (Garcke) to 63.6% on *Desmodium tortuosum* (Sw.) DC. Researchers have noted that *B. tabaci* mortality tends to be highest in the first instar, particularly the crawler stage [30,33,34]. Key predators of whiteflies, including coccinellids and predatory mites, feed preferentially on *B. tabaci* eggs and early instars [35]. The same leaf characteristics that influence host acceptance and oviposition by whiteflies, such as type and density of trichomes, degree of pubescence, waxiness, and leaf texture, can also effect searching and the degree of mortality inflicted by predators and parasitoids [36,37]. Additional studies are needed to determine whether differential survival of nymphs on distinct weed hosts is due to differences in host suitability, differences in predation rates, including host feeding by parasitoids, or a combination of factors.

Unlike other studies which have evaluated colony-reared *B. tabaci* host choice and development on weeds under controlled conditions [28,30,38], we measured egg and nymph densities produced by naturally occurring whitefly populations under field conditions. Our data indicate that oviposition by *B. tabaci* can be high on *A. theophrasti*, *E. fosbergii* and *C. obtusifolia*, and that these weeds can support significant *B. tabaci* populations.

Compared to these hosts, *Bidens alba* supported moderate to low densities of whitefly nymphs, but at least in 2012, comparatively high levels of parasitism. Our findings are consistent with those of Stansly *et al.* [20] who measured up to 52% parasitism on the closely related *B. pilosa*. Whether *B. alba* has a primarily positive or negative effect on managing *B. tabaci* in the region may depend on its as yet undetermined role as a reservoir for TYLCV.

*Amaranthus retroflexus* has been described as a poor and possibly even a non-host of *Bemisia tabaci* in other studies [18,28,30,38]. While egg densities were relatively high in 2012 on *A. retroflexus*, they were very low in 2013, and nymph densities were consistently very low, not surpassing 6 nymphs per 100 cm^2^ (11 June 2012 and 17 June 2013). Percent parasitism on *A. retroflexus* was 7.9 in 2012, not significantly different from percent parasitism on *A. theophrasti*, *E. fosbergii*, and *S. americanum*, although these weed hosts had significantly higher nymph densities than *A. retroflexus* on most weeks in 2012 and 2013. As a poor host of *B. tabaci* which supports levels of parasitism similar to levels observed on heavily infested weeds, *A. retroflexus* may play a mitigating role in the development of *B. tabaci* populations. Papayiannis *et al.* [13] detected TYLCV in field collected *A. retroflexus* on Cyprus. The influence of *A. retroflexus* on whitefly-related pest problems in Florida may depend on its as yet undetermined role in the epidemiology of TYLCV.

In addition to being a host of *B. tabaci*, *S. americanum* is a host of pepper weevil (*Anthonomus eugenii* Cano) [39]. Its congener, *Solanum nigrum* L., has been identified as a host of TYLCV in several studies [40]. Stansly *et al.* [20] observed 26.5% parasitism of *B. tabaci* on *S. americanum*, which was higher than what we observed in either year. Stansly *et al.* [20] recorded 34.9% parasitism *B. tabaci* on *S. acuta*. We observed 28.8% parasitism in *S. acuta* during the week of July 17, 2012, and 17.5% parasitism overall for the season. *Sida acuta* has been identified as a host of *Tomato yellow leaf curl Tanzania virus* [41].

As its common name implies, the sweetpotato whitefly has a long documented association with plants in the genus *Ipomoea* [42,43,44] and other genera in the Convolvulaceae [10,12]. Whitefly-transmitted geminiviruses of *Ipomoea* are distributed globally [45]. We consistently detected moderate or low levels of eggs and nymphs on *I. lacunosa* during each season of study. *Ipomoea lacunosa* germinated and grew rapidly when planted from seed in 2012, but senesced just as rapidly, with no parasitism recorded that year. Percent parasitism was generally low on *I. lacunosa* in 2013, with the highest level (9.5%) measured on 17 June.

Densities of *B. tabaci* eggs and nymphs were consistently low on *S. exaltata* in 2012, and whiteflies were extremely rare on this host in 2013 with the result that sampling of this was abandoned that year. Leaflets on *S. exaltata* are small—8 mm × 3.5 cm or less [46], providing a limited substrate for whitefly to oviposit on or for the completion of nymphal development.

### 4.2. Parasitoids

Consistent with other surveys of whitefly parasitoids in Florida, we recovered primarily *Encarsia* spp. parasitoids and a low number of *Eretmocerus* [23,47,48]. Of the *Encarsia* species that could be identified to species, 14% were *E. sophia*, and 12.5% were *E. tabacivora*. Further investigation is required to determine if differences in percent parasitism on weeds was influenced by leaf characteristics of different hosts. For example, McAuslane *et al.* [47] determined that leaf hairiness influenced percentage parasitism of whitefly on soybean (*Glycine max* L (Merr.)) in Florida, with *E. nigricephala* and *E. transvena* (a synonym of *E. sophia*) more common on glabrous than hirsute soybean, while the opposite was true of *E. pergandiella* and *Er*. nr. *californicus*. Tests on collards (*Brassica oleracea* var. *acephala* L.) in Florida demonstrated that while waxiness did not affect parasitism by *Eretmocerus* sp., more than 4.5 times as many *E. pergandiella* individuals emerged from collards with glossy leaves *versus* those with normal wax [49].

Although *B. tabaci* densities were much lower in 2013 than 2012, a similar pattern with regard to weed colonization was revealed each year. Oviposition on *A. theophrasti*, *C. obtusifolia* and *E. fosbergii* indicated that these three weed species can support high densities of *B. tabaci* under favorable conditions. By contrast *B. tabaci* densities on *B. alba*, *A. retroflexus* and *I. lacunosa* were consistently moderate or low, and numbers on *S. exaltata* were negligible each season. Among these weed species, *B. alba* tends to dominate uncultivated areas in parts of west central Florida to a greater extent than other species. The high numbers of parasitized nymphs observed on some dates in 2012 confirm that some weeds can support significant parasitism of *B. tabaci* and provide alternate parasitism sites for key parasitoids of *B. tabaci* in Florida, primarily *Encarsia* and *Eretmocerus* spp.

Our data indicate that the proportion of *B. tabaci* completing development from egg to adult on most species was often low. Additional studies are needed to reveal the primary factors affecting survival of immature *B. tabaci* on different weed hosts. Weeds with characteristics that are moderately favorable for whiteflies, such as *B. alba* and *A. retroflexus*, but suitable for significant levels of parasitism, may play a positive role in mitigating over-summering populations of whitefly. However it must first be confirmed that these and other weeds do not play a significant role in the epidemiology of *Tomato yellow leaf curl* and other plant viruses in central and south Florida.
